# Antioxidant and Prophylactic Effects of *Delonix elata* L., Stem Bark Extracts, and Flavonoid Isolated Quercetin against Carbon Tetrachloride-Induced Hepatotoxicity in Rats

**DOI:** 10.1155/2014/507851

**Published:** 2014-06-02

**Authors:** Pradeepa Krishnappa, Krishna Venkatarangaiah, Venkatesh, Santosh Kumar Shivamogga Rajanna, Rajesh Kashi Prakash Gupta

**Affiliations:** Department of Post Graduate Studies and Research in Biotechnology, Kuvempu University, Shankaraghatta, Karnataka 577 451, India

## Abstract

*Delonix elata* L. (Ceasalpinaceae), is widely used by the traditional medical practitioners of Karnataka, India, to cure jaundice, and bronchial and rheumatic problems. The objective of this study was to screen the *in vitro* antioxidant and hepatoprotective activity of the stem bark extracts against CCl_4_-induced liver damage in rats. Among different stem bark extracts tested, the ethanol extract (DSE) has shown significant *in vitro* antioxidant property in radicals scavenging, metal chelating, and lipid peroxidation inhibition assays. HPLC analysis of the DSE revealed the presence of known antioxidant molecules, namely, gallic acid, ellagic acid, coumaric acid, quercetin, and rutin. Bioassay-guided fractionation of DSE has resulted in the isolation and characterization of quercetin. DSE and quercetin have shown significant prophylactic effects by restoring the liver function markers (AST, ALT, ALP, serum bilirubin, and total protein) and antioxidant enzymes (SOD, CAT, GPx, and GST). These results were proved to be hepatoprotective at par with silymarin and well supported by the histological observations of liver sections with distinct hepatic cells, and mild degree of fatty change and necrosis. The results indicated that the DSE and quercetin were significant for prophylactic activity against CCl_4_-induced liver damage in rats. This activity could be attributed to the antioxidant constituents in the DSE and hence justified the ethnomedicinal claims.

## 1. Introduction

Liver is a vital organ of human body which performs detoxification of the exogenous xenobiotics, drugs, viral infections, and chronic alcoholism. While performing several detoxifications, liver undergoes stress, leading to liver diseases ending in liver damage and serious health problems and death [1]. Liver damage is a widespread pathology which in most cases involves oxidative stress and is characterized by a progressive evolution from steatosis to chronic hepatitis, fibrosis, cirrhosis, and hepatocellular carcinoma [2]. In recent years, attention has been focused on the role of biotransformation of chemicals into highly reactive metabolites that initiate cellular toxicity. Carbon tetrachloride- (CCl_4_-) induced hepatotoxicity in animal model has been widely used to investigate hepatoprotective effect of the natural compounds [3, 4]. As oxidative stress plays a central role in liver pathologies and their progression, the use of antioxidants has been proposed as therapeutic agents, as well as drug coadjuvants, to counteract liver damage.

Despite advancements in modern medicine, hepatoprotective drugs are quite limited which include cholagogues, choleretics, immunoglobulin, corticosteroids, penicillamine, trimethobenzamide, chenodiol [5]. The prolonged usage of remedies available in modern medicine is associated with severe side effects. The development or identification of new molecules effective in treating or preventing hepatic damage remains a challenge in the field of drug development [6].

There is widespread recognition that, indigenous drugs used traditionally by ethnic tribes or societies across the globe can provide respite to patients with hepatic disorders. As a result a conscious effort is employed to screen indigenous drugs used conventionally in different parts and regions of the world, especially India and China. Plant origin drugs which exhibit hepatoprotective and antioxidant activities were isolated from many species. A well-known potential hepatoprotective drug, silymarin was isolated from* Silybum marianum* [7].

*Delonix elata* Linn. (family: Caesalpinaceae) is a deciduous tree and sparsely distributed in the dry forests of India. Traditional medical practitioners residing in the villages of Chitradurga district, Karnataka, India, used the leaves and stem bark extracts for curing jaundice, hepatic disorders, and bronchial and rheumatic problems. The plant is also reported for problems like pain and stiffness of the joints, especially the knees [8]. Leaves are used for the treatment of bronchitis in infants, fever, malaria, flatulence, and paralysis or as carminative [9]. In our previous study, leaves extract of* D. elata* has shown remarkable antinociceptive activity [10] and antibacterial activity [11]. Leaf extract has been screened for anti-inflammatory activity [12]. In view of these reports and the ethnomedical claims, the present investigation has been undertaken to evaluate the* in vitro* antioxidant and prophylactic effects of stem bark extracts of* D. elata* against CCl_4_ intoxicated liver damage in rat.

## 2. Materials and Methods

### 2.1. Chemicals

Silymarin was purchased from Micro labs Bangalore, India; carbon tetrachloride, petroleum ether, chloroform, ethanol, butylated hydroxyl anisole (BHA), acrylamide, N,N-methylene bisacrylamide, sulfanilamide, sodium nitroprusside (SNP), trichloroacetic acid (TCA), and Folin-Ciocalteu reagent were purchased from Merck Ltd., Mumbai, India. Quercetin, thiobarbituric acid (TBA), 2,2-diphenyl-1-picrylhydrazyl (DPPH), nicotinamide adenine dinucleotide (NADH), ferrozine, nitroblue tetrazolium disodium salt (NBT), phenazine methosulfate (PMS), and O-dianisidine were obtained from Sigma-Aldrich (St. Louis, MO, USA). Gallic acid, ascorbic acid, ferrous chloride, and potassium persulfate were procured from HiMedia (India). All the chemicals used were of analytical grade. Water was purified using Milli-Q system from Millipore (Millipore, Bedford, MA, USA). Diagnostic test kits for the assay of liver function markers were purchased from Robonik (India) Pvt. Ltd., Mumbai, India.

### 2.2. Preparation of Extracts

The stem barks of* D. elata* were collected from the Chitradurga, Karnataka, India, in October 2010. Taxonomic authentication of the plant was done by Dr. Manjunatha, compared with the voucher specimen deposited at Kuvempu University [13]. The plant materials were air-dried in shade, pulverized, and stored in airtight containers. The powdered material (500 g) was refluxed successively with the solvents petroleum ether, chloroform, and ethanol in a Soxhlet extractor for 48 h. The extracts were filtered by Whatman filter paper number 1 and the filtrates were concentrated in vacuum under reduced pressure on a rotary evaporator (Buchi, Switzerland). The yields of petroleum ether, chloroform, and ethanol extracts of stem bark were 0.53%, 0.75%, and 9.67%, respectively.

### 2.3. Qualitative Phytochemical Analysis

The petroleum ether (DSP), chloroform (DSC), and ethanol (DSE) sequential extracts of stem bark were tested qualitatively for presence of various phytochemical groups using standard tests [14, 15].

### 2.4. Quantitative Estimation of Crude Extracts 

#### 2.4.1. Determination of Total Phenol Content

Total phenol content was measured by the method described by Chang et al. [16]. 1 mL of each extract (200 *μ*g) was mixed with Folin-Ciocalteu reagent (2 mL) (diluted 1 : 10, v/v) followed by the addition of 2 mL of sodium carbonate (7.5%, w/v) and allowed to stand for 90 min at room temperature and absorbance was measured against the blank at 750 nm using spectrophotometer (Systronics, PC based double beam spectrophotometer 2202). Total phenol content of the extracts was expressed in terms of gallic acid equivalent (GAE, *μ*g of dry mass).

#### 2.4.2. Determination of Total Flavonoid

Total flavonoid content was determined according to the modified method of Zhishen et al. [17]. 5 mL of extract (200 *μ*g) was mixed with 300 *μ*L of 5% sodium nitrite and 300 *μ*L of 10% aluminum chloride followed by the addition of 2 mL of 1 M sodium hydroxide. After the incubation of reaction mixture at room temperature for 6 min, the volume was made up to 10 mL by adding 2.4 mL of Millipore water. Absorbance was measured at 510 nm against the blank. Total flavonoid content of the extract was expressed in terms of quercetin equivalents (QE, *μ*g of dry mass).

#### 2.4.3. HPLC-UV Analysis

Phenolic acids and flavonoids present in stem bark extracts were analyzed by HPLC (Model LC-10ATVP. Shimadzu Corporation, Kyoto, Japan) on a reversed-phase Shimpak C18 column (5 *μ*m, 250 mm × 4.6 mm). Phenolic content in the extracts was detected using octadecylsilyl-silica gel as stationary phase. Solvent system consisting of [A] phosphoric acid : water (0.5 : 99.5, v/v) and [B] acetonitrile was used as mobile phase at a flow rate of 1 mL min^−1^. Phenolic acid standards such as gallic acid, p-coumaric acid, ellagic acid, hydroxybenzoic acid, and vanillic acids were employed for identification of phenolic acids present in DSC and DSE by comparing the retention time under similar experimental conditions. The detector used for analysis was UV detector at 220 nm. Flavonoid content in the extracts was detected using octadecylsilyl-silica gel as stationary phase. Solvent system consisting of methanol, water, and phosphoric acid (50 : 49.6 : 0.4, v/v) was used as mobile phase at a flow rate of 0.5 mL min^−1^. Flavonoids namely, rutin, quercetin, myricetin, kaempferol, and luteolin were used as references.

#### 2.4.4. Isolation and Characterization of Flavonoid Compound

The DSE (15 g) was subjected to silica gel column chromatography (60 × 4 cm, 60–120 mesh, 200 g), eluted with a stepwise gradient of chloroform and chloroform-methanol combination (9 : 1, 8 : 2, 7 : 3, and 6 : 4). A total of 325 fractions (10 mL each) were eluted. Fractions 114–119 yielded a residue of about 0.76 g. This residue was further purified by preparative TLC (silica gel) using the solvent system chloroform-methanol in the ratio of 7 : 3 to afford yellow compound (207 mg). Characterization of the isolated compound was performed by subjecting it to qualitative analysis followed by IR, ^1^H-NMR, ^13^C-NMR, and mass spectral studies.

### 2.5. Determination of* In Vitro* Antioxidant Activity

*In vitro* antioxidant activity of the stem bark sequential solvent extracts (DSP, DSC, and DSE) was determined by performing the following experiments.

The total antioxidant and total reductive capability of stem bark extracts were determined by the methods of Prieto et al. and Oyaizu [18, 19], respectively. The DPPH free radical-scavenging activity was measured according to the procedure described by Braca et al. [20]. The ability of DSP, DSC, and DSE to scavenge hydroxyl radical was determined by the method of Klein et al. [21]. Superoxide anion radical scavenging activity was measured according to the method of Nishikimi et al. [22]. Nitric oxide radical scavenging assay was determined using the method of Marcocci et al. [23]. Metal chelating activity of DSP, DSC, and DSE was measured by adapting the method of Dinis et al. [24]. The method of Halliwell and Gutteridge [25] was used to determine the lipid peroxidation inhibition assay. The antioxidant activity of BHT, ascorbic acid, EDTA, and curcumin was determined for comparison. The IC_50_ (the concentration required to scavenge 50% of radical) value was calculated using the formula: IC_50_ = [(∑C/∑I) × 50], where ∑C is the sum of extracts concentrations used for testing and ∑I is the sum of percentage of inhibition at different concentrations.

### 2.6. Acute Toxicity Study

Acute toxicity study was conducted for the stem bark extracts (DSC and DSE) and quercetin by the Up and Down procedure [26]. DMSO (1% v/v) was used as a vehicle to suspend the extracts and administered orally. Animals were observed individually at least once during the first 30 min after dosing, periodically during the first 24 h (with special attention given during the first 4 h), and daily thereafter, for a total of 14 days for changes in their behavioural pattern and mortality.

### 2.7. Prophylactic Effect of Stem Bark Extracts against CCl_4_-Induced Liver Damage in Rats

#### 2.7.1. Experimental Design

Wistar albino rats of either sex, weighing about 180–200 g were used for the study. Animals were housed at (25 ± 1)°C and humidity of 55–60% in the Department of Biotechnology, Kuvempu Univeristy, Shimoga, Karnataka, India. They were fed with standard commercial pellet diet (Sai Durga feeds and foods, Bangalore) and water* ad libitum* during the experiment. The Institutional Ethical Committee (Registration Number: NCP/IAEC/CL/13/12/2010-11) permitted the study.

Rats were divided into eight groups consisting of six animals in each group. Group-1 served as normal control and received 1% (v/v) DMSO (1 mL/kg of body weight, p.o); group-2 (toxic control) received 50% CCl_4_ in olive oil (1 mL/kg of body weight, i.p); groups-3 and -4 received DSC (100 and 300 mg kg^−1^ p.o, resp.); groups-5 and -6 received DSE (100 and 300 mg kg^−1^ p.o, resp.); group-7 received quercetin (20 mg kg^−1^); group-8 received standard drug silymarin (25 mg kg^−1^ p.o) once a day. Treatment duration was 15 days and all the groups received the intraperitoneal dose of CCl_4_ after every 72 h [4].

At the end of the experimental period, animals were sacrificed. Blood was collected and serum was separated. The liver tissues were excised and used for the assays of liver function markers and antioxidant enzymes.

#### 2.7.2. Estimation of Liver Function Marker Metabolites

Liver damage was assessed by estimating serum marker enzymes such as ALT, AST, and ALP using biochemical analyzer (Robonik India Pvt. Ltd., New Mumbai). The results were expressed as units litre^−1^ (UL^−1^). The levels of cholesterol, triglycerides (TG), total bilirubin, and total protein were estimated in the serum of experimental animals using assay kits obtained from the Robonik India Pvt. Ltd., New Mumbai.

#### 2.7.3. Estimation of Antioxidant Enzymes Activity

Liver tissue (10%) was homogenized in ice cold normal saline and centrifuged at 4,000 rpm for 5 min. The supernatant was used for the following assays.

The activity of SOD was assayed by measuring its ability to inhibit the photochemical reduction of nitroblue tetrazolium (NBT) using the method of Beauchamp and Fridovich [27] and the results have been expressed as units (U) of SOD mg^−1^ protein. The catalase (CAT) activity was determined by the method of Aebi [28]. Glutathione peroxidase (GPx) activity was measured by the method of Mohandas et al., [29]. Glutathione-S-transferase (GST) activity was determined by the method of Warholm et al., [30]. The activities of these enzymes are expressed as nmol min^−1 ^mg^−1^ of protein.

#### 2.7.4. *In Vivo* Lipid Peroxidation (LPO) Assay

Malondialdehyde (MDA) is one of lipid peroxidation products determined by the method of Ohkawa et al., [31]. In brief, 0.5 mL of the 10% homogenate was mixed with 100 *μ*L of FeCl_3_ (0.2 mM, 2 mL) and reaction mixture (0.25 N HCl containing 15% TCA, 0.30% TBA, and 0.05% BHA), incubated at 80°C for 1 h, cooled, and then centrifuged at 1,500 rpm. The supernatant was collected. Lipid peroxidation products were estimated by measuring the concentration of thiobarbituric acid reaction substances (TBARS) in fluorescence at 530 nm.

#### 2.7.5. Histopathology of Liver Tissue

The liver tissue was washed with normal saline and kept in 10% formaldehyde buffer for 18 h. The tissues were dehydrated in graded (50–100%) ethanol, followed by clearing in xylene. Paraffin (56–58°C) embedding was done at 58 ± 1°C for 4 h, followed by paraffin block preparation. Paraffin sections of 5 *μ*m were taken using a rotary microtome. The sections were deparaffinised with alcohol xylene series, stained with haematoxylin-eosin, and mounted in DPX with a cover slip and histological changes were observed under microscope [32].

### 2.8. Statistical Analysis

Results are expressed as mean ± S.E.M. The statistical analysis was carried out using one-way ANOVA followed by Duncan's test. The differences in values at *P* < 0.05 or *P* < 0.01 were considered as statistically significant. Statistical analysis was performed by GraphPad Prism 5 software.

## 3. Results

### 3.1. Phytochemical Screening of Stem Bark Extracts

The qualitative phytochemical screening of stem bark extracts of* D. elata* showed the presence of alkaloids, flavonoids, terpenoids, saponins, steroids, tannins, proteins, and carbohydrates. The quantitative estimation of phenolics and flavonoids contents in stem bark extracts is shown in Table 1. Among them, DSE possessed the highest total phenolics (77.75 ± 0.05 *μ*g of dry extract GAE) and flavonoid contents (75.33 ± 0.67 *μ*g of dry extract QE), while, the petroleum ether extract (DSP) comprised the lowest total phenolics (7.85 ± 0.01 *μ*g of dry extract GAE) and flavonoid contents (0.16 ± 0.35 *μ*g of dry extract QE).

### 3.2. HPLC-UV Analysis

**Figure 1:**
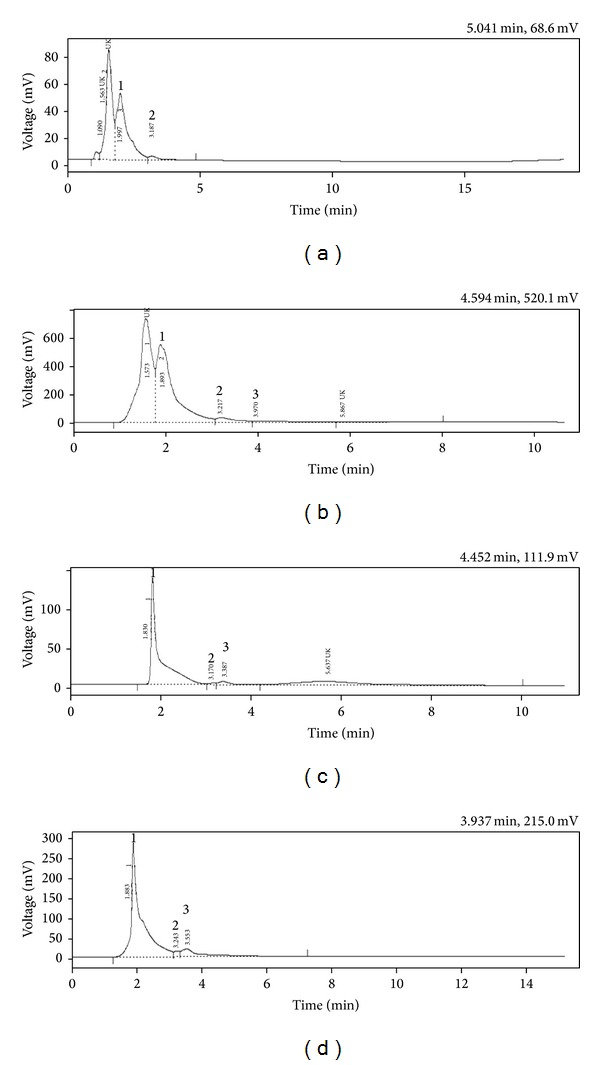
(a) HPLC chromatogram of DSC for phenolic acids (1-gallic acid; 2-coumaric acid; UK-unknown); (b) HPLC chromatogram of DSE for phenolic acids (1-gallic acid; 2-coumaric acid; 3-ellagic acid; UK-unknown); (c) HPLC chromatogram of DSC for flavonoids (1-rutin; 2-quercetin; 3-myricetin; UK-unknown); (d) HPLC chromatogram of DSE for flavonoids (1-rutin; 2-quercetin; 3-myricetin; UK-unknown).

The DSC and DSE were subjected to HPLC analysis for the characterization of phenolic acids and flavonoids (Table 2). The HPLC analysis of DSC revealed the presence of gallic acid, coumaric acid, and two unknown phenol compounds with retention time of 1.99, 3.18, 1.09, and 1.56 min (Figure 1(a)). The DSE contains gallic acid, coumaric acid, ellagic acid, and two unknowns with retention time of 1.89, 3.2, 3.97, 1.573, and 5.86 min, respectively (Figure 1(b)). Similarly, the HPLC-UV spectral peaks of DSC (Figure 1(c)) and DSE (Figure 1(d)) at 350 nm with the analysis of retention time of standard flavonoids showed the presence of flavonoid compounds, rutin, quercetin, and myricetin.

### 3.3. Isolation and Characterization of Quercetin

**Figure 2:**
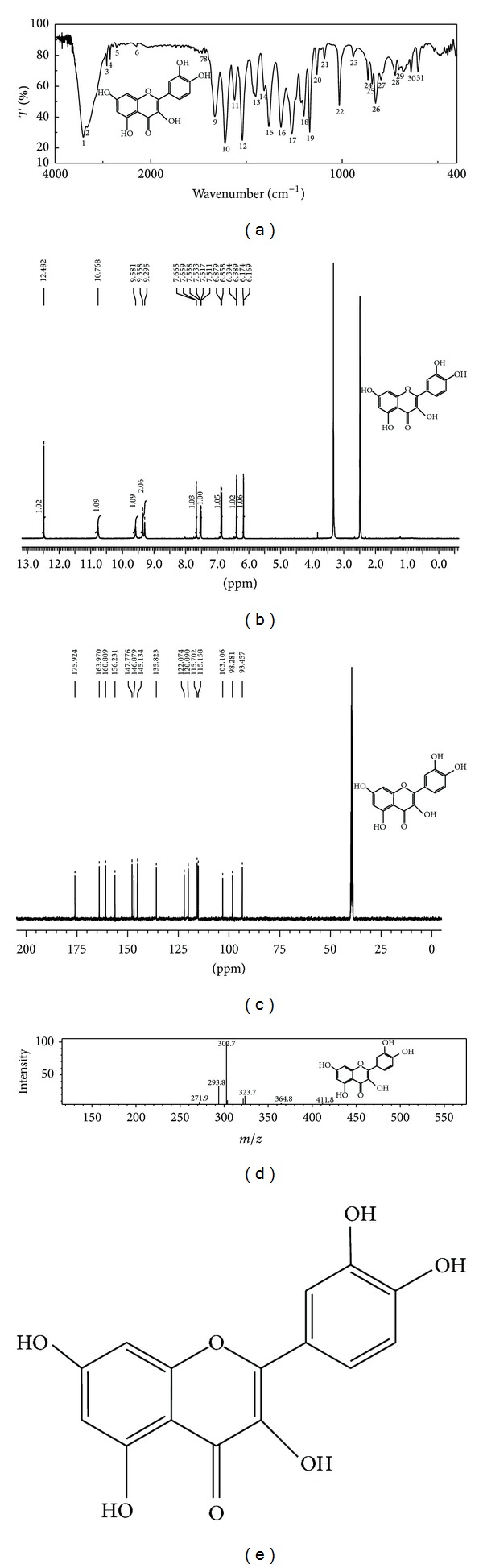
(a) IR spectrum; (b) ^1^H NMR spectrum; (c) ^13^C NMR spectrum; (d) Mass spectrum; (e) Structure of quercetin.

The DSE was eluted through silica gel column chromatography using the solvent system chloroform-methanol in the ratio of 7 : 3 and it yielded a yellow amorphous compound with melting point 314–316°C. In qualitative group testing (lead acetate solution test, alkaline reagent test, ferric chloride test, and Shinoda's test), it gave positive results for flavonoids. The IR (Figure 2(a)) spectrum showed the peak values at (KBr. *V*_max⁡_  cm^−1^) = 3410.49 (br-OH), 1611.23 (C=O); 1H-NMR (Figure 2(b)) peak values with (DMSO-d6): *δ* ppm = 6.1 (2H, Ar-H), 6.3 (2H, Ar-H), 7.5 (5H, Ar-OH), 9.5 (1H, Ar-H) and 13C-NMR (Figure 2(c)) peak values with (DMSO-d6): *δ* ppm = 135.823 (1C, Ar-C), 145.134 (1C, Ar-C), 175.924 (1C, C=O), 93.457 (1C, RCH_2_OH), 98.281 (1C, RCH_2_OH), 103.106 (1C, RCH_2_OH), 115.158 (1C, RCH_2_OH), 115.702 (1C, RCH_2_OH), MS: *m*/*z* = 302.7 (M^+^) (Figure 2(d)). The molecular formula of the isolated compound was deduced as C_15_H_10_O_7_ and it is characterized as quercetin (Figure 2(e)).

### 3.4. Determination of* In Vitro* Antioxidant Activity

**Figure 3:**
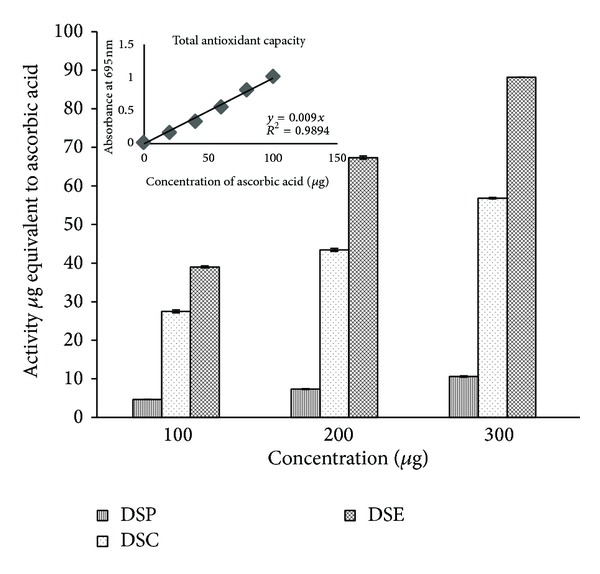
Total antioxidant capacity of sequential solvent extracts of stem bark of* D. elata.*

**Figure 4:**
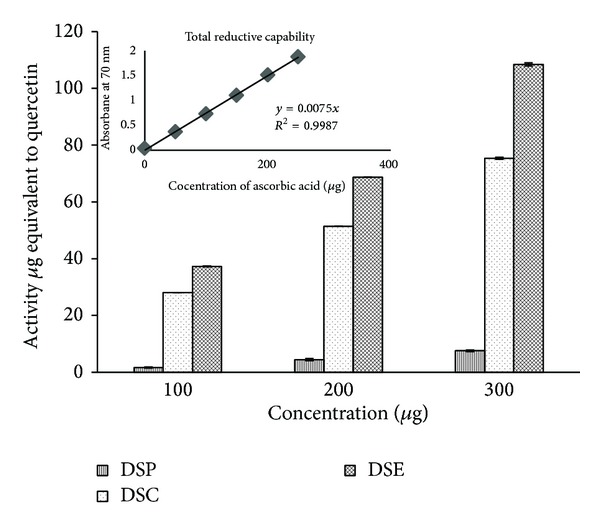
Total reductive capability of sequential solvent extracts of stem bark of* D. elata*.

Stem bark extracts were found to be considerably different in their total antioxidant activities. Among the three extracts, the total antioxidant potentialities of DSE was highest (88.2 ± 0.01 *μ*g AAE) followed by the effect of DSC (56.83 ± 0.17 *μ*g AAE, ascorbic acid equivalents). The DSP exhibited the least antioxidant effect (10.61 ± 0.16 *μ*g AAE) as shown in Figure 3. Among the three concentrations of the extracts tested (100, 200 and 300 *μ*g mL^−1^), the total antioxidant activity was increased with increasing concentrations of the extracts. In reductive capability assay also the DSE showed greater reductive capabilities (108.44 ± 0.54 *μ*g QE, quercetin equivalents) and its capacity increased with the increase of concentrations as shown in Figure 4. The radical scavenging potentialities of the three extracts of the stem bark tested at three different concentrations are shown in Table 3. Among the three extracts tested, DSE showed significant radical scavenging activity compared to DSC while DSP showed the least radical scavenging activity. The IC_50_ values of the extracts for antioxidant activity are shown in Table 4. DSE exhibited the highest scavenging potentiality in superoxide radical scavenging, (IC_50_; 167.24 ± 0.12 *μ*g mL^−1^), nitric oxide radical scavenging assay (IC_50_; 306.48 ± 1.1 *μ*g mL^−1^), and hydroxyl radical scavenging assay (IC_50_; 423.43 ± 0.38 *μ*g mL^−1^) whereas DSP expressed the least activity in superoxide radical scavenging, nitric oxide radical scavenging assay, and hydroxyl radical scavenging assay. In DPPH radical scavenging assay, the potency of DSE was more (IC_50_; 82.88 ± 0.18 *μ*g mL^−1^) than the standard BHT (IC_50_; 85.26 ± 0.41 *μ*g mL^−1^).

The effect of sequential solvent extracts of stem bark on the metal chelating and inhibition of lipid peroxidation is also summarized in Tables 3 and 4. The DSE chelated ferrous ion with minimum IC_50_ value as compared to other extracts in a dose dependent manner. Similarly, inhibition of lipid peroxidation by DSE was more potent (IC_50_; 939.15 ± 9.61 *μ*g mL^−1^) than other extracts tested.

Since, DSP exhibited very poor activity in the* in vitro* antioxidant assays, it was not considered for hepatoprotective activity against CCl_4_-induced liver damage.

### 3.5. Acute Toxicity Studies

Acute toxicity studies revealed that, the animals administered with DSC and DSE at concentration of 3,000 mg kg^−1^ b.w. showed 50% of lethality (LD_50_). One tenth of the LD_50_ dose, that is 300 mg kg^−1^ b.w., was considered as safe dose. LD_50_ of the quercetin was found to be 200 mg kg^−1^ b.w., and hence 20 mg kg^−1^ is considered as safe dose for oral administration.

### 3.6. Prophylactic Effect of Stem Bark Extracts

The intoxication of CCl_4_ to the rats had resulted in a marked increase in the levels of liver function serum markers, namely, AST (561.97 ± 9.64 U L^−1^), ALT (161.90 ± 2.37 U L^−1^), ALP (400.10 ± 4.91 U L^−1^), total bilirubin (14.07 ± 1.04 mg/dL^−1^), triglyceride (215.97 ± 5.48 mg dL^−1^), total cholesterol (274.00 ± 3.51 mg dL^−1^) and in the decrease of total proteins level (5.84 ± 0.09 g dL^−1^) as compared to the control group treated with only vehicle (1% DMSO). On the contrary, the increased levels of these liver function markers were brought down nearer to normalcy due to the ameliorative effect of the stem bark extracts. Significant hepatoprotective activity was noticed in the animals treated with the DSE at the dosage of 300 mg kg^−1,^ namely, AST (320.47 ± 3.58 U L^−1^), ALT (89.93 ± 10.94 U L^−1^), ALP (281.42 ± 6.28 U L^−1^), total bilirubin (0.05 ± 0.01 mg dL^−1^), triglyceride (176.93 ± 1.65 mg dL^−1^), cholesterol (186.00 ± 3.21 mg dL^−1^), and total protein (7.22 ± 0.19 mg dL^−1^). In animals treated with DSC, reduction of toxic effect of CCl_4_ was reduced significantly as compared to toxic control group. However, the prophylactic effect was less as compared to DSE and quercetin. The isolated constituent quercetin exhibited significant protection. The hepatoprotective effect of DSE, DSC, and quercetin was comparatively evaluated with the standard drug silymarin as shown in Table 5.

The oxidative stress markers in the liver homogenates revealed that intoxication of rats with CCl_4_ significantly decreased the activities of oxidative stress marker enzymes in liver like SOD (5.16 ± 0.07 U mg^−1^), CAT (214.6 ± 0.7 nmol min^−1 ^mg^−1^), GPx (87.14 ± 0.35 nmol NADPH min^−1^ mg^−1^), and GST (209.58 ± 1 nmol min^−1 ^mg^−1^) as compared to the toxic control group (SOD, 12.47 ± 0.38 U mg^−1^; CAT, 484.82 ± 3.17 nmol min^−1 ^mg^−1^; GPx, 163.72 ± 0.19 nmol min^−1^ mg^−1^; and GST, 422.7 ± 1.9 nmol min^−1 ^mg^−1^) (Table 6). In addition, a 2-fold increase in levels of MDA was noticed in CCl_4_ intoxicated rat as compared to normal animals.

Among the two extracts tested for* in vivo* antioxidant activity, the liver of DSE (300 mg kg^−1^) administered rats showed significant ameliorative effect by elevating the reduced levels of SOD (7.91 ± 0.01 U mg^−1^), CAT (364.55 ± 0.45 nmol min^−1 ^mg^−1^), GPx (137.94 ± 0.44 nmol NADPH min^−1^ mg^−1^), and GST (337.15 ± 0.45 nmol min^−1 ^mg^−1^) levels. The increased level of MDA was also restored (0.66 ± 0.01 nmol mg^−1^). The restoration levels of oxidative stress marker enzymes were significant in quercetin and moderate in the group treated with DSC (300 mg kg^−1^ b.w.). The values are shown in Table 6.

### 3.7. Histopathological Examination of Liver Tissue

**Figure 5:**
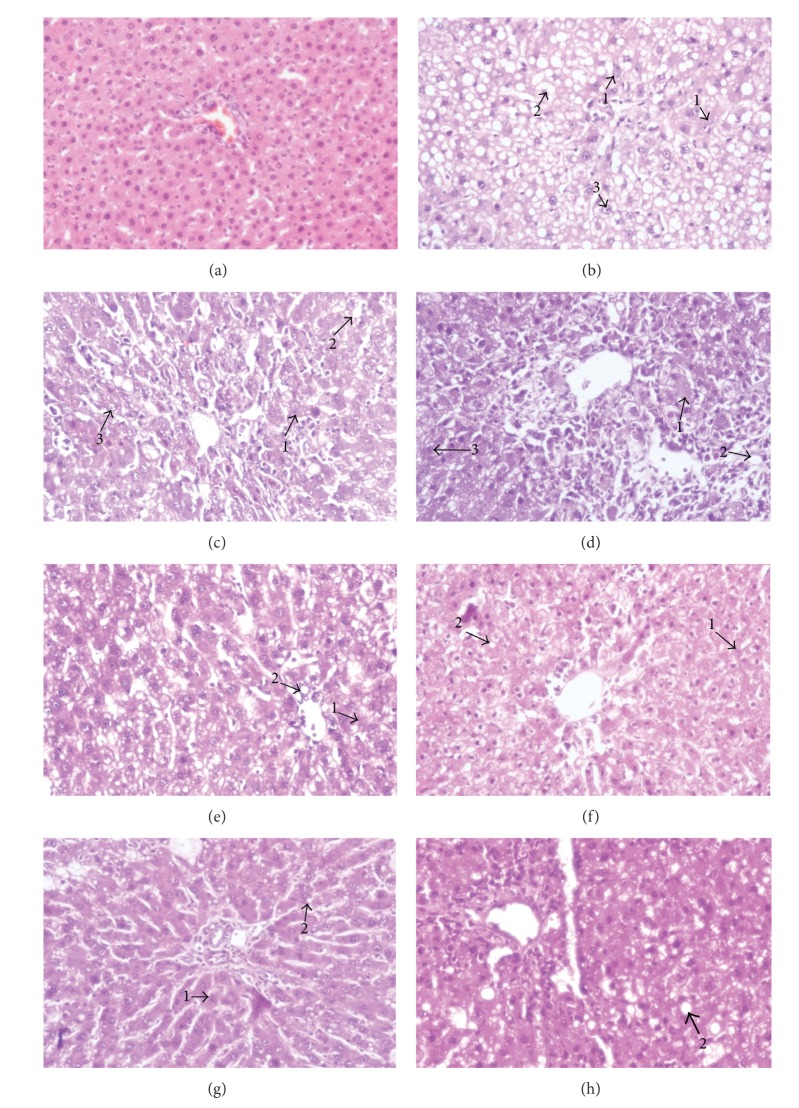
Histopathology of liver tissues. (a) Liver section of normal control rats showing normal architecture; (b) Liver section of CCl_4_ treated rats showing massive fatty changes, cellular necrosis, vacuolization, and ballooning degeneration; (c) Liver section of rats treated with CCl_4_ and 100 mg kg^−1^ of DSC showing mild fatty vacuolation and cellular necrosis; (d) Liver section of rats treated with CCl_4_ and 300 mg kg^−1^ of DSC showing cellular regeneration, mild degree of cell necrosis, and fatty vacuolation; (e) Liver section of rats treated with CCl_4_ and 100 mg kg^−1^ of DSE showing few fatty vacuoles, restoration of hepatocytes, and lesser necrosis; (f) Liver section of rats treated with CCl_4_ and 300 mg kg^−1^ of DSE showing minimal inflammatory cellular infiltration and almost near normal liver architecture; (g) Liver section of rats treated with CCl_4_ and quercetin showing recovery from the toxic effect with normal arrangement of hepatocytes with little evidence of fatty vacuoles and cellular necrosis (h) Liver histology of animals treated with silymarin showing normal histology with least parenchymal injury. (1-cellular necrosis; 2-vacuolization; 3-ballooning degeneration).

The histological profile of liver sections of the control animals showed normal hepatic architecture with well-preserved cytoplasm, prominent nucleus, central vein, and compact arrangement of hepatocytes without fatty lobulation (Figure 5(a)). The liver sections of CCl_4_ treated animals showed hydropic changes in centrilobular hepatocytes with cell necrosis surrounded by neutrophils. Congestion of the central vein and sinusoids was seen with acute and chronic inflammatory cells infiltrating sinusoids mainly in the central zone. The midzonal and periportal hepatocytes showed vacuolization and fatty change (steatosis) which includes the intracellular accumulation of neutral fat (Figure 5(b)). The hepatocytes are distended with fat vacuoles due to increased deposition of intracellular lipids in liver section of DSC (100 mg kg^−1^ b.w.) administered animals (Figure 5(c)). In the liver sections of rats administered with DSC (300 mg kg^−1^ b.w.) showed mild fatty changes and mild sinusoidal congestion (Figure 5(d)). Animals administered with DSE (100 mg kg^−1^ b.w) exhibited significant liver protection against CCl_4_-induced liver damage. It is evident by the presence of hepatic cords with moderate fatty change and few inflammatory cells, and absence of necrosis (Figure 5(e)). The sections of liver taken from the animals treated with DSE (300 mg kg^−1^ b.w.) and quercetin (Figures 5(f) and 5(g) resp.) showed the normal hepatic architecture with presence of very few inflammatory cells and cell necrosis. Liver section of the rats treated with silymarin showed the presence of normal hepatic cords with few numbers of inflammatory cells and necrosis (Figure 5(h)).

## 4. Discussion

Oxidative stress is the unbalance between reactive oxygen and nitrogen species (ROS/RNS) production and the antioxidant defense and plays a pivotal role in different pathophysiological conditions [25]. It has been suggested that an intake of a rich in antioxidant diet is inversely associated with the risk to develop some pathologies like liver diseases [33, 34]. Thus, attention has been paid on the antioxidant capacity of natural products and many of the indigenous medicinal plants were screened for antioxidant properties.* D. elata* traditionally have been in use by the people residing in the villages of Chitradurga (Karnataka), India, as a potent pain relieving and hepatoprotective medicine. To study the potential antioxidant health-protecting effects of* D. elata* and to consider the complexity involved in their* in vivo* mechanisms of action, a single* in vitro* chemical method was not enough to authenticate the antioxidant properties. It is necessary to apply more than one* in vitro* chemical-based assay that evaluates various mechanisms, such as, prevention of chain initiation, binding with transition metal ion catalysts, decomposition of peroxides, prevention of continued hydrogen abstraction, and reducing the capacity and radical scavenging ability of the extracts towards ROS/RNS. Hence, oxidative stress related assays of interest were adopted to authenticate the antioxidant potency of* D. elata* stem bark extracts.

The model of scavenging DPPH (1,1-diphenyl-2-picrylhydrazyl) radical is a widely used method to evaluate the free radical scavenging activities of antioxidants. In the DPPH assay, the antioxidants are able to reduce the stable DPPH radical (purple) to the nonradical form DPPH-H (yellow) [35]. The DSE showed significant radical scavenging activity in the DPPH scavenging assay among the tested extracts of* D. elata* and it was more effective than the standard reference BHT. The DPPH scavenging ability of the DSE is attributed to their hydrogen donating ability. Among ROS, hydroxyl radical is one of the potent reactive oxygen species in the biological system that reacts with polyunsaturated fatty acid (PUFA) moieties of cell membrane phospholipids and causes damage to cell [36]; superoxide anion is a weak oxidant, it ultimately produces powerful and dangerous hydroxyl radicals and singlet oxygen [37]. Nitric oxide (NO) is a free radical of RNS, produced in the mammalian cells. The excess production of nitric oxide is involved in oxidative stress and also associated with several diseases like adjuvant arthritis, inflammation, cancer, and so forth [38].

In the present investigation, DSE was found to scavenge the OH^−^, O_2_^−^ and NO-free radicals significantly and in dose dependent manner. It has been observed that the stem bark of* D. elata* contains an amazing diversity of secondary metabolites. One of the most important groups of these metabolites is phenolic compounds. Among stem bark extracts tested, DSE possessed high concentration of total phenolic and flavonoid content. In recent years there has been growing interest in antioxidant properties of phenolic compounds. Antioxidant action of phenolic compounds is due to their high tendency to chelate metals. Phenolics possess hydroxyl and carboxyl groups able to bind particularly to iron and copper [39]. They may inactivate iron ions by chelating and additionally suppressing the superoxide-driven Fenton reaction, which is believed to be the most important source of ROS [40, 41] and DSE has shown good metal chelating ability. Metal ions decompose lipid hydroperoxide (LOOH) by the hemolytic cleavage of the O–O bond and give lipid alkoxyl radicals, which initiate free radical chain oxidation. Phenolic antioxidants inhibit lipid peroxidation by trapping the lipid alkoxyl radical. This activity depends on the structure of the molecules and the number and position of the hydroxyl group in the molecules [42]. Flavonoids are group of phenolic compounds that can directly scavenge molecular species of active oxygen: superoxide, hydrogen peroxide, hydroxyl radical, singlet oxygen, or peroxyl radical. Their antioxidant action resides mainly in their ability to donate electrons or hydrogen atoms [43]. Polyphenolic flavonoids are occurring ubiquitously in medicinal plants [44]. Many flavonoids are reported as strong scavengers of the reactive oxygen species due to the presence of phenolic hydroxyl groups [45].

To know the possible phenolics and flavonoids present in the extracts, HPLC-UV analysis has been carried out and the reports have shown the presence of well-known flavonoids and phenolic acids, which are proved as good antioxidants by several investigators [46–49]. The phytochemical investigation has been carried to isolate bioactive principle from the promising extract, DSE. The result of the present investigation revealed that DSE was bestowed with pharmacologically active quercetin. Quercetin is considered to be a strong antioxidant due to its ability to scavenge free radicals and bind transition metal ions. These properties of quercetin allow it to inhibit lipid peroxidation [50, 51]. Similarly,* in vitro* radical scavenging activity* was* carried out in* Mesua ferrea* [44],* Launaea procumbens* [52], and* Swertia chirayita* [53].

Reducing power, reflecting the electron donation capacity, is one of the most important indicators of antioxidant activity of bioactive compounds [54]. By donating electrons, antioxidant substances are able to block radical chain reaction by converting reactive oxygen species to more stable products. In present study, DSE reduced the Fe^3+^/ferricyanide complex to the ferrous form in a dose dependent manner. Similarly, reducing power activity has been assessed in* Talinum triangulare* [3],* Cordia macleodii* [55], and* Fumaria* species [56].

Carbon tetrachloride is metabolized by cytochrome P4502E1 (CYP2E1) to the trichloromethyl (^•^CCl_3_) and trichloromethyl peroxy (^•^OOCCl_3_) radicals are mainly associated with CCl_4_-induced hepatic damage [57]. The covalent binding of these radicals to cellular macromolecules, with preference to polyunsaturated fatty acids (PUFA) of the cellular membranes leads to generation of the fatty acid free radicals, which initiate autocatalytic lipid peroxidation, ultimately resulting in the loss of membrane integrity and leakage of microsomal enzymes.

This process is evidenced by an elevation in the serum marker enzymes AST, ALT, and ALP after CCl_4_ administration in rats. Carbon tetrachloride also induces cellular hypomethylation, leading to inhibition of protein synthesis (possibly through ribosomal RNA hypomethylation) and defects in lipid and protein metabolism [58]. Thus in the present study, significant elevation in the levels of serum marker enzymes and significant decrease in the total protein level were noticed in the animals treated with CCl_4_. The administration of DSE (300 mg kg^−1^ b.w) and quercetin (20 mg kg^−1^ b.w.) reduced toxic effect of CCl_4_ by restoring the levels of serum marker enzymes to the normalcy. DSC (300 mg kg^−1^ b.w.) has shown significant results when compared to the toxic control group. However, the prophylactic effect of DSC was less compared to DSE (300 mg kg^−1^ b.w.). The antioxidant enzymes like SOD, CAT, GPx, and GST play an important role in defense mechanisms against the harmful effects of reactive oxygen species (ROS) and free radicals in biological systems. The fact that DSE and quercetin treatment increased SOD, CAT, GPx, and GST levels back to their normal control levels indicated that DSE and quercetin may prevent the peroxidation of lipids by CCl_4_, whereas, DSC also significantly increased the antioxidant enzymes level but the effect was comparatively less.

Increased serum levels of AST, ALT, and ALP are indicators of liver damage but are not specific to liver damage. Elevated ALP is used in the diagnosis of hepatobiliary disease and bone disease; elevated serum AST level is observed during myocardial damage [59]. An additional parameter is required to confirm the presence of liver injury. Numerous studies have demonstrated the occurrence of lipid peroxidation following carbon tetrachloride exposure by detection of by-products of lipid peroxidation such as MDA. These by-products can form protein and DNA adducts and may contribute to hepatotoxicity [60]. Natural antioxidants, like glutathione, are capable of preventing the lipid peroxidation reaction. When antioxidants are depleted, opportunities for lipid peroxidation are enhanced [61]. CCl_4_ is capable of generating highly reactive free radicals, inhibiting GSH synthesis, increasing MDA levels, and impairing antioxidant defense systems in humans and experimental animals.

In order to trace the possible mechanism by which stem bark extract prevents hepatic damage caused by CCl_4_ and to examine the presence of oxidative stress in CCl_4_ treated rat livers, investigations on lipid peroxidation were carried out in the liver homogenate. In the present study, CCl_4_ treated animals have shown elevated levels of MDA content in liver, whereas the DSE and quercetin administered animals significantly reduced the levels of MDA towards normalcy as compared to toxic control group. DSC also significantly reduced the MDA levels compared to toxic control animals. However, prophylactic effect was less compared to DSE and quercetin. Hence it is suggested that possible mechanism of prophylactic effect of the DSE, DSC, and quercetin against CCl_4_ toxicity is due to their antioxidant effect.

Histopathological observations of liver sections of rats treated with carbon tetrachloride in mineral oil exhibited a significant number of ballooned hepatocytes and inflammatory cells at 12 h and progressive, massive centrilobular steatosis, inflammation, and cellular necrosis at 18–48 h due to covalent binding of ^•^CCl_3_ and ^•^OOCCl_3_ radicals of CCl_4_ to cellular macromolecules (nucleic acid, protein, and lipid), impairing crucial cellular processes such as lipid metabolism [62]. DSE and its constituent, quercetin, exhibited significant prophylactic effect by the action of antioxidant activity and it is clearly observed in histological observations of liver sections with distinct hepatic cells, sinusoidal spaces, a central vein, and a mild degree of fatty change, necrosis, and lymphocyte infiltration almost comparable to the silymarin treated group. Similar investigations were carried out by several investigators [63–65]. There are earlier reports stating the role of quercetin against hepatotoxicity and oxidative stress [66–69].

## 5. Conclusion

The results revealed that the antioxidant effects of ethanol extracts of stem bark of* D. elata*, showed consistent and concentration-dependent antioxidant activities, as well as a significant protection against CCl_4_-induced liver injury. The presence of these activities could be attributed to the bioactive principle quercetin or synergic effect of the constituents present in it. The result of this investigation strongly supports the ethnomedical claims of* D. elata*. Further studies are required to understand the mechanism of action of DSE that is responsible for hepatoprotective and antioxidant effects.

## Figures and Tables

**Table 1:** Total phenolic and flavonoid contents of *D. elata* stem bark extracts.

Samples	Phenolic content (*μ*g GAEs/mg extract)	Flavonoid content (*μ*g QEs/mg extract)^b^
DSP	7.85 ± 0.01	0.16 ± 0.35
DSC	45.71 ± 0.25	61.50 ± 1.16
DSE	77.75 ± 0.05	75.33 ± 0.67

Note: Values expressed are means ± SEM of three parallel measurements. GAEs-gallic acid equivalents; QEs-quercetin equivalents.

**Table 2:** Quantitative estimation of *D. elata* stem bark extracts for phenolic acids and flavonoids by HPLC.

Phenolic acids and flavonoids	DSC (mg g^−1^)	DSE (mg g^−1^)
Phenolic acids		
Gallic acid	392.00	474.05
Coumaric acid	3.422	17.60
Ellagic acid	—	106.50
Hydroxybenzoic acid	—	—
Vanillic acid	—	—
Flavonoids		
Rutin	144.29	245.30
Quercetin	30.60	161.20
Myricetin	11.08	—
Kaempferol	—	—
Luteolin	—	—

Note: DSC-stem bark chloroform extract of *D. elata* and DSE-stem bark ethanol extract of *D. elata*.

**Table 3:** *In vitro* antioxidant activity of *D. elata* stem bark extracts.

Serial number	Activity	Concentration of extracts in *μ*g	% of inhibition		
			DSP	DSC	DSE
1	DPPH radical scavenging activity	50	2.49 ± 0.11	15.73 ± 0.37	34.36 ± 0.01
		100	5.22 ± 0.22	27.49 ± 0.05	60.91 ± 0.33
		150	8.57 ± 0.56	47.29 ± 0.31	85.71 ± 0.05

2	Superoxide radical scavenging activity	100	1.27 ± 0.17	20.65 ± 0.35	37.22 ± 0.21
		200	4.50 ± 0.28	44.36 ± 0.21	58.60 ± 0.01
		300	6.44 ± 0.09	64.43 ± 0.43	83.56 ± 0.34

3	Hydroxyl radical scavenging assay	100	2.53 ± 0.07	10.83 ± 0.28	22.2 ± 0.01
		250	5.58 ± 0.09	23.48 ± 0.41	39.13 ± 0.32
		500	10.37 ± 0.13	47.52 ± 0.05	77.34 ± 0.18

4	Nitric oxide radical scavenging activity	100	2.53 ± 0.07	10.83 ± 0.28	22.20 ± 0.01
		250	5.58 ± 0.09	23.48 ± 0.41	39.13 ± 0.32
		500	10.37 ± 0.13	47.52 ± 0.05	77.34 ± 0.18

5	Metal chelating	1000	0.47 ± 0.03	7.51 ± 0.39	27.29 ± 0.29
		2000	2.80 ± 0.05	15.43 ± 0.34	49.36 ± 0.16
		3000	4.46 ± 0.03	22.12 ± 0.09	65.44 ± 0.16

6	Lipid peroxidation inhibition	500	1.62 ± 0.26	12.33 ± 0.09	19.6 ± 0.38
		1000	3.42 ± 0.03	23.62 ± 0.18	35.30 ± 0.59
		1500	4.58 ± 0.14	31.15 ± 0.26	51.58 ± 0.12

The results shown are averages of three independent experiments; values are mean ± SEM.

**Table 4:** IC_50_ values of stem bark extracts of *D. elata* for *in vitro* antioxidant activity.

Serial number	Activity	DSP (*μ*g)	DSC (*μ*g)	DSE (*μ*g)	Standard (*μ*g)
1	DPPH radical scavenging	922.1 ± 6.05	165.72 ± 0.2	82.88 ± 0.18	85.26 ± 0.41 (BHT)
2	Superoxide radical scavenging	2459.02 ± 6.04	231.78 ± 1.78	167.24 ± 0.12	101.13 ± 1.0 (Ascorbic acid)
3	Hydroxyl radical scavenging	2852.72 ± 32.55	681.75 ± 6.62	423.43 ± 0.38	125.66 ± 1.2 (BHT)
4	Nitric oxide radical scavenging	2300.14 ± 2.86	519.4 ± 1.18	306.48 ± 1.10	98.34 ± 0.74 (Curcumin)
5	Metal chelating	38861.15 ± 21.35	6659.84 ± 13.87	2111.23 ± 4.42	31.52 ± 0.2 (EDTA)
6	Lipid peroxidation inhibition	10409.9 ± 29.66	1490.42 ± 3.66	939.15 ± 9.61	—

The results shown are averages of three independent experiments; values are mean ± SEM.

BHT-vutylated hydroxytoluene; EDTA-ethylenediaminetetra acetic acid.

**Table 5:** Prophylactic effect of *D. elata* stem bark extracts and quercetin on restoration of liver function markers in CCl_4_ intoxicated rats.

Groups	ALP (U/L)	AST (U/L)	ALT (U/L)	Total Bilirubin (mg/dL)	Total Cholesterol (mg/dL)	Triglyceride (mg/dL)	Total protein (g/dL)
Control 1% DMSO	223.77 ± 5.85**	201.20 ± 3.84**	61.75 ± 3.34**	0.04 ± 0.01**	150.00 ± 2.00**	173.50 ± 2.75**	8.28 ± 0.15**
CCl_4_ (50%) 1 mL kg^−1^	400.10 ± 4.91	561.97 ± 9.64	161.90 ± 2.37	14.07 ± 1.04	274.00 ± 3.51	215.97 ± 5.48	5.84 ± 0.09
DSC 100 mg kg^−1^ + CCl_4_	346.3 ± 1.05**	413.84 ± 1.37**	127.02 ± 0.63**	0.09 ± 0.01**	250.60 ± 0.52**	199.28 ± 0.94^ns^	6.94 ± 0.20**
DSC 300 mg kg^−1^ + CCl_4_	308.50 ± 7.55**	356.60 ± 3.88**	107.70 ± 7.34**	0.076 ± 0.01**	225.00 ± 3.00**	197.70 ± 0.82^ns^	6.95 ± 0.27**
DSE 100 mg kg^−1^ + CCl_4_	332.08± 0.80**	390.94 ± 0.17**	110.98 ± 0.26**	0.07 ± 0.01**	212.30 ± 0.70**	189.44 ± 0.12**	7.12 ± 0.05**
DSE 300 mg kg^−1^ + CCl_4_	281.42 ± 6.28**	320.47 ± 3.58**	89.93 ± 10.94**	0.05 ± 0.01**	186.00 ± 3.21**	176.93 ± 1.65**	7.22 ± 0.19**
Quercetin 20 mg kg^−1^ + CCl_4_	281.37 ± 1.47**	330.56 ± 2.0**	97.81 ± 3.5**	0.05 ± 0.01**	192 ± 1.03**	181. 21 ± 2.31**	7.4 ± 0.22**
Silymarin 25 mg kg^−1^ + CCl_4_	241.87 ± 1.88**	316.13 ± 5.98**	84.63 ± 2.26**	0.03 ± 0.01**	186.00 ± 3.06**	162.23 ± 1.12**	7.86 ± 0.16**

Each value represents mean ± S.E.M. of 6 animals. Symbols represent statistical significance. **P < 0.0001 and ns-not significant as compared to CCl_4_ toxic control.

**Table 6:** Effect of stem bark extracts and quercetin on antioxidant liver markers in CCl_4_ intoxicated rats.

Groups	GPx (nmol NADPH min^−1^ mg^−1^ protein)	GST (nmol min^−1^ mg^−1^ of protein)	CAT (nmol min^−1^ mg^−1^ of protein)	SOD (Umg^−1^ of protein)	MDA (nmol mg^−1^ of protein)
Control 1% DMSO	163.72 ± 0.19**	422.7 ± 1.9**	484.82 ± 3.17**	12.47 ± 0.38**	0.64 ± 0.01**
CCl_4 _(50%) 1 mL kg^−1^	87.14 ± 0.35	209.58 ± 1.0	214.6 ± 0.7	5.16 ± 0.07	1.11 ± 0.01
DSC 100 mg kg^−1^ + CCl_4_	94.38 ± 0.29^ns^	295 ± 0.11**	253.02 ± 1.42**	6.36 ± 0.02**	0.92 ± 0.01**
DSC 300 mg kg^−1^ + CCl_4_	106.51 ± 0.81**	312.5 ± 1.74**	289.34 ± 0.33**	6.68 ± 0.03**	0.85 ± 0.01**
DSE 100 mg kg^−1^ + CCl_4_	104.65 ± 0.15*	304.44 ± 2.17**	306.22 ± 1.89**	7.17 ± 0.06**	0.83 ± 0.01**
DSE 300 mg kg^−1^ + CCl_4_	137.94 ± 0.44**	337.15 ± 0.45**	364.55 ± 0.45**	7.91 ± 0.01**	0.66 ± 0.01**
Quercetin 20 mg kg^−1^ + CCl_4_	134.47 ± 0.22**	340.58 ± 0.37**	348.80 ± 2.12**	7.63 ± 0.1**	0.64 ± 0.01**
Silymarin 25 mg kg^−1^ + CCl_4_	140.14 ± 1.11**	362.2 ± 1.0**	362.85 ± 0.05**	8.73 ± 0.14**	0.64 ± 0.01**

Each value represents mean ± S.E.M. of 6 animals. Symbols represent statistical significance. **P < 0.0001 and ns-not significant as compared to CCl_4_ toxic control.
